# Supplementary material for: Predicting depression among men who have sex with men in Ghana using machine learning algorithms
Source: PLOS Ment Health. 2025 Nov 20;2(11):e0000485. doi: 10.1371/journal.pmen.0000485 (PMC12798198; doi:10.1371/journal.pmen.0000485)
Supplement: S1 Table — Each category represents a group of related variables used to assess key psychosocial domains such as perceived stress, social isolation, behavioral risk, and stigma related to same-sex behavior and gender non-conformity among MSM in Ghana. (DOCX) [file pmen.0000485.s001.docx]

S1 Table: *Summary of psychological and social constructs included in the model. Each category represents a group of related variables used to assess key domains such as perceived stress, social isolation, behavioral risk, and stigma related to same-sex behavior and gender non-conformity among MSM in Ghana.*

| **Category** | **Variable(s)** | **Values** | **Scoring description** | **Encoding** | **Source** |
| --- | --- | --- | --- | --- | --- |
| Depression | PHQ1: Little interest or pleasure in doing things  PHQ2: Feeling down, depressed or hopeless | 0=Not at all, 1=Several days, More than half days= 2 and Nearly every day =3 | Ordinal, summed for total | one-hot encoding | [1] |
| Perceived Stress | PSS1: In the last month, how often have you been upset because of something that happened unexpectedly?  PSS2: In the last month, how often have you felt that you were unable to control the important things in your life?  PSS3: In the last month, how often have you felt nervous and “stressed”?  PSS4: In the last month, how often have you dealt successfully with irritating life hassles?  PSS5: In the last month, how often have you felt that you were effectively coping with important changes that were occurring in your life?  PSS6: In the last month, how often have you felt confident about your ability to handle your personal problems?  PSS7: In the last month, how often have you felt that thing were going your way?  PSS8: In the last month, how often have you found that you could not cope with all the things that you had to do?  PSS9: In the last month, how often have you been able to control irritations in your life?  PSS10: In the last month, how often have you felt that you were on top of things?  PSS11: In the last month, how often have you been angered because of things that were outside of your control?  PSS12: In the last month, how often have you found yourself thinking about things that you have to accomplish?  PSS13: In the last month, how often have you been able to control the way you spend your time?  PSS14: In the last month, how often have you felt difficulties were piling up so high that you could not overcome them? | Never=0, Almost never=1, Sometimes=2, Fairly often=3 and Often =4 | \|  \| \| --- \|  \| Sum or mean (after reverse-coding select items) \| \| --- \| | one-hot encoding | [2] |
| External Social Isolation | ExtSocialIso1: How often in the previous two weeks have you spent time together with family?  ExtSocialIso1: How often in the last week did you meet face to face with friends and relatives living outside your  household? | Every day=4  Never=0 | sum | one-hot encoding | [3],[4],[5] |
|  | ExtSocialIso3: Do you have anyone with whom you can discuss intimate and personal matters? | a) Yes b) No c) Refusal d) Don’t know e) No answer | sum | one-hot encoding |  |
|  | ExtSocialIso4: If you were in trouble, do you have relatives or friends you can count on to help, such as financial assistance? | a) Yes  b) No | sum | one-hot encoding |  |
|  | ExtSocialIso5: If someone does a favour for me, I am ready to return it  ExtSocialIso6 : I go out of my way to help somebody who has been kind to me before  ExtSocialIso7: I am ready to undergo personal costs to help somebody who helped me before | 1= does not apply to me at all  7= applies to me perfectly | sum | one-hot encoding |  |
|  | ExtSocialIso8: In the last 12 months have you done any volunteer activities through or for an organization? | a) Yes  b) No | sum | one-hot encoding |  |
| Internal Social Isolation | IntSocialIso1: In general, how satisfied or unsatisfied are you with your:   - Friends - Family - Neighbourhood/town/community/   Spiritual or religious Spouse or partner | 1 = Very satisfied  2 = Fairly satisfied  3 = Not very satisfied  4 = Not at all satisfied; 99 = No Answer | sum | one-hot encoding | [6],[7],[8] |
|  | IntSocialIso2: How strongly do you feel you belong to your immediate community/neighbourhood?  Indicate how often you feel the way described in each of the following statements.  IntSocialIso3: How often do you feel that you are ‘in tune’ with the people around you?  IntSocialIso4: How often do you feel that no one really knows you well?  IntSocialIso5: How often do you feel you can find companionship when you want it?  IntSocialIso6: How often do you feel that people are around you but not with you?  Response structure: | 1 = Very strongly  2 = Fairly strongly  3 = Not very strongly  4 = Not at all strongly  5 = Don't know | sum | one-hot encoding |  |
|  | IntSocialIso7: Generally speaking, would you say that most people can be trusted or that you can’t be too careful in dealing with people? | 1 = Never  2 = Rarely  3 = Sometimes  4 = Often  Note: Questions 1 and 3 must be reversed before scoring (i.e., 1=4, 2=3, 3=2, 4=1). | sum | one-hot encoding |  |
|  | IntSocialIso8: In general, do you agree or disagree with the following statements?  IntSocialIso9: Most people in this village/neighbourhood are willing to help if you need it.  IntSocialIso10: In this village/neighbourhood, one has to be alert or someone is likely to take advantage of you. | 1 = Agree strongly  2 = Agree somewhat  3 = Neither agree or disagree  4 = Disagree somewhat  5 = Disagree strongly | sum | one-hot encoding |  |
|  | IntSocialIs13: If you lost a wallet that contained 800 cedis, and it was found by a neighbour, how likely is it to be returned with the money in it? | 1 = Very likely  2 = Somewhat likely  3 = Somewhat unlikely  4 = Not at all likely? | sum | one-hot encoding |  |
| Behavioural Risk | BRScale1. I tend to bounce back quickly after hard times  BRScale2.I have a hard time making it through stressful situations  BRScale3.It does not take me long to recover from a stressful situation.  BRScale4.It is hard for me to snap back when something bad happens.  BRScale5.I usually come through difficult times with little trouble.  BRScale6.I tend to take a long time to get over set-backs in my life. | 1 = Strongly disagree  2=Disagree  3=Neutral  4=Agree  5=Strongly agree | Reverse coding (where needed) + sum | one-hot encoding | [9] |
| Same-Sex Behavior Stigma | StigmaSSB1. How often have you heard that homosexuals are not normal?  StigmaSSB2. How often have you felt that your homosexuality hurt and embarrassed your family?  StigmaSSB3. How often have you been made fun of or called names for being homosexual?  StigmaSSB4. How often have you been hit or beaten up for being homosexual?  StigmaSSB5. How often have you had to pretend that you are not homosexual in order to be accepted?  StigmaSSB6. How often has your family not accepted you because of your homosexuality?  StigmaSSB7. How often have you lost your friends because of your homosexuality?  StigmaSSB8. How often have you been kicked out of school for being homosexual?  StigmaSSB9. How often have you lost a place to live for being homosexual?  StigmaSSB10. How often have you lost a job or career opportunity for being homosexual? | Never=0, Once or twice=1, A few times=2 and Many times=3 | sum | one-hot encoding | [10],[11] |
| Gender Non-Conformity Stigma | StigmaGNC1. Do people consider you feminine by looking at you, your mannerisms, or your behaviour?  StigmaGNC2. How often have you heard that men with feminine mannerisms are not normal?  StigmaGNC3. How often have you felt that your feminine mannerisms and behaviour hurt and embarrassed your family?  StigmaGNC4. How often have you had to pretend that you were more masculine in order to be accepted?  StigmaGNC5. How often have you been hit or beaten up for your feminine mannerisms and behaviour?  StigmaGNC6. How often has your family not accepted you for feminine mannerisms and behaviour?  StigmaGNC7. How often have you lost straight friends because of your feminine mannerisms and behaviour?  StigmaGNC8. How often have you been verbally harassed by the police for your feminine mannerisms and behaviour?  StigmaGNC9. How often have you been physically harassed by the police for your feminine mannerisms and behaviour?  StigmaGNC10. How often have you been sexually harassed by the police for your feminine mannerisms and behaviour?  StigmaGNC11. How often have you lost a place to live because of your feminine mannerisms and behaviour?  StigmaGNC12. How often have you lost a job or career opportunity because feminine mannerisms and behaviour?  StigmaGNC13. How often have been made fun or called names because of your feminine mannerisms and behaviour? | Never=-0, Once or twice=1, A few times=2 and Many times=3 | sum | one-hot encoding | [11],[12] |
| Sense of Community Scale | SCS1. I can get what I need in this from my network of friends.  SCS2. My network of friends helps me fulfill my needs.  SCS3. I feel like a member of my network of friends.  SCS4. I belong in my network of friends.  SCS5. I have a say about what goes on in my network of friends.  SCS6 People in my network of friends are good at influencing each other.  SCS7. I feel connected to my network of friends.  SCS8. I have a good bond with the people in my network of friends. | Strongly Disagree=1, Disagree=2, Neutral=3, Agree=4 and 5=Strongly Agree | sum | one-hot encoding | [13] |

References (for S1 Table)

[1] Kroenke K, Spitzer RL, Williams JB. The Patient Health Questionnaire-2: validity of a two-item depression screener. *Medical Care*. 2003;41(11):1284–1292.

[2] Cohen S, Kamarck T, Mermelstein R. A global measure of perceived stress. *Journal of Health and Social Behavior*. 1983;24(4):385–396.

[3] Department for Environment, Food & Rural Affairs. *Survey of Public Attitudes and Behaviours Towards the Environment: 2011*. UK Government; 2011.

[4] European Social Survey Round 5. *ESS5 - 2010 Documentation Report*. European Social Survey; 2010.

[5] German Socio-Economic Panel (SOEP). *Data for Research on Family, Household, and Individual Behavior*. German Institute for Economic Research (DIW); 2016.

[6] Cummins RA. The Domains of Life Satisfaction: An Attempt to Order Chaos. *Social Indicators Research*. 1996;38:303–328.

[7] UK Department for Communities and Local Government. *Citizenship Survey: 2010–2011*.

[8] Russell DW. UCLA Loneliness Scale (Version 3): Reliability, validity, and factor structure. *Journal of Personality Assessment*. 1996;66(1):20–40.

[9] Smith BW, Dalen J, Wiggins K, et al. The Brief Resilience Scale: Assessing the ability to bounce back. *International Journal of Behavioral Medicine*. 2008;15(3):194–200.

[10] Meyer IH. Prejudice, social stress, and mental health in lesbian, gay, and bisexual populations: conceptual issues and research evidence. *Psychological Bulletin*. 2003;129(5):674–697.

[11] Puckett JA, Woodward EN, Mereish EH, Pantalone DW. Parental rejection following sexual orientation disclosure: Impact on internalized homophobia, social support, and mental health. *LGBT Health*. 2015;2(3):265–269.

[12] McMillan DW, Chavis DM. Sense of community: A definition and theory. *Journal of Community Psychology*. 1986;14(1):6–23.

[13] McMillan, DW., & Chavis, D M. (1986). Sense of community: A definition and theory. Journal of Community Psychology, **14**(1), 6–23.
https://doi.org/10.1002/1520-6629(198601)14:1<6::AID-JCOP2290140103>3.0.CO;2-I
