# Supplementary material for: Predicting depression among men who have sex with men in Ghana using machine learning algorithms
Source: PLOS Ment Health. 2025 Nov 20;2(11):e0000485. doi: 10.1371/journal.pmen.0000485 (PMC12798198; doi:10.1371/journal.pmen.0000485)
Supplement: S1 Fig — The models include Logistic Regression, Decision Tree, Random Forest, Naïve Bayes, and K-Nearest Neighbour classifiers, used to predict the severity of depression among MSM in Ghana. (DOCX) [file pmen.0000485.s002.docx]

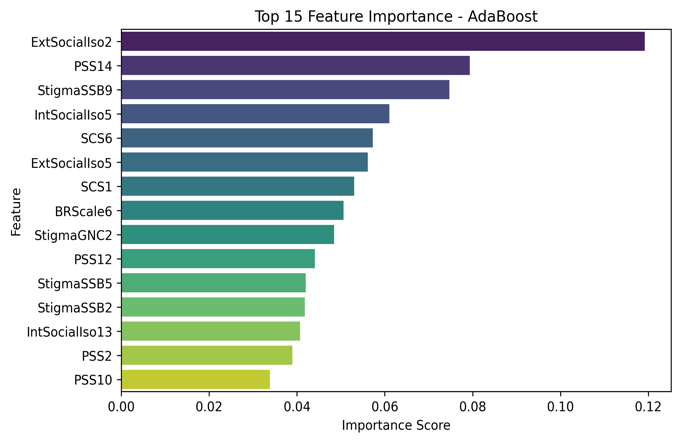

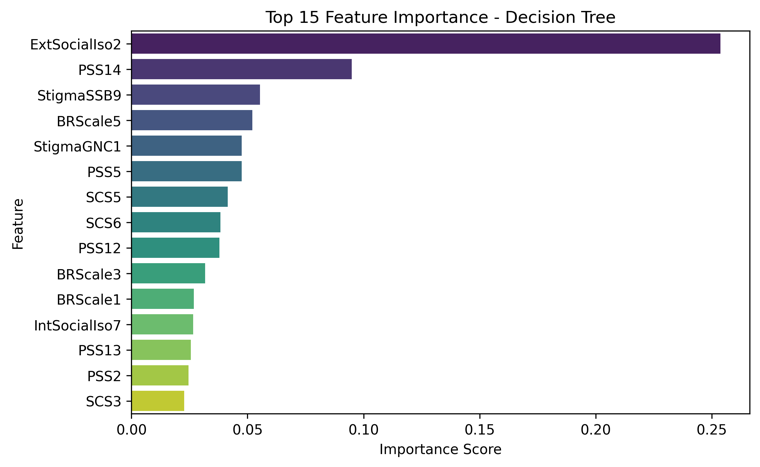

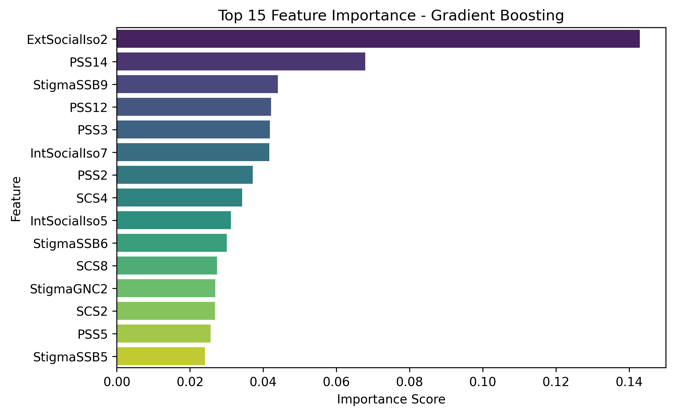

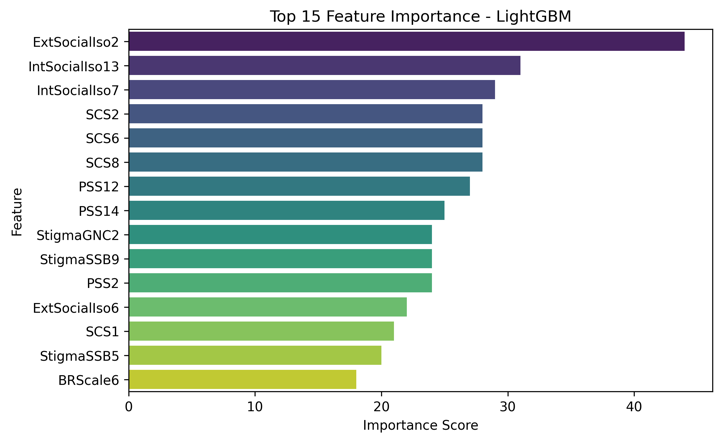

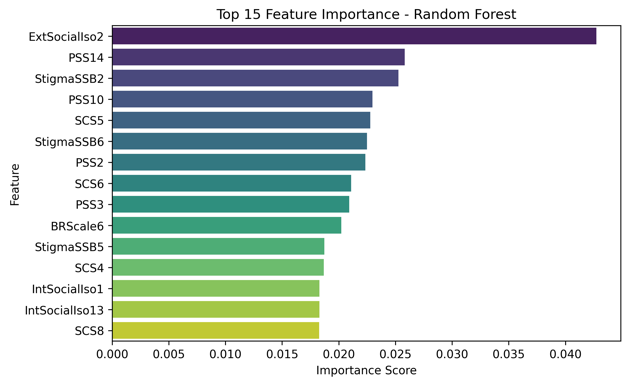

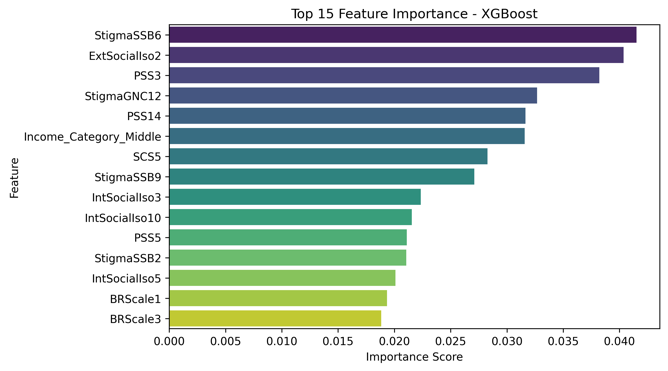


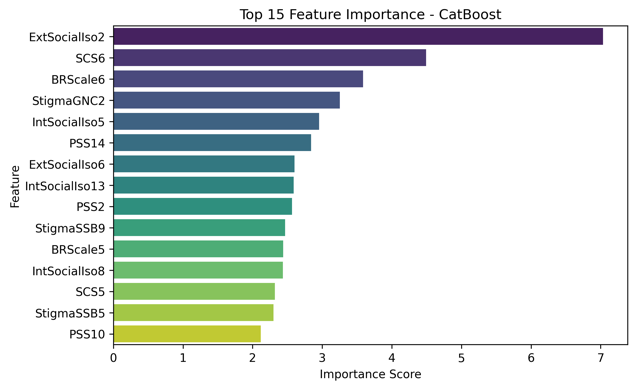


*S2 Fig: Top 15 most important features in predicting depression among MSM in Ghana using machine learning classifiers. External social isolation (ExtSocialIso2), perceived stress (PSS14), and stigma related to same-sex behavior (StigmaSSB9) emerged as leading predictors across both models.*
