# Supplementary material for: Predicting depression among men who have sex with men in Ghana using machine learning algorithms
Source: PLOS Ment Health. 2025 Nov 20;2(11):e0000485. doi: 10.1371/journal.pmen.0000485 (PMC12798198; doi:10.1371/journal.pmen.0000485)
Supplement: S2 Table — Random Forest achieved the highest mean accuracy (80.36%), followed by CatBoost (77.36%) and XGBoost (76.09%), demonstrating strong generalization performance across folds. (DOCX) [file pmen.0000485.s003.docx]

*S2 Table.* Five-fold cross-validation accuracy scores for seven tree-based classification models predicting depression among MSM in Ghana. Random Forest achieved the highest mean accuracy (80.36%), followed by CatBoost (77.36%) and XGBoost (76.09%), indicating strong generalization performance across folds.

| **Model** | **Fold 1** | **Fold 2** | **Fold 3** | **Fold 4** | **Fold 5** | **Mean Accuracy** |
| --- | --- | --- | --- | --- | --- | --- |
| Decision Tree | 0.6596 | 0.6809 | 0.766 | 0.7447 | 0.6304 | 0.6963 |
| Random Forest | 0.8298 | 0.8511 | 0.8085 | 0.6809 | 0.8478 | 0.8036 |
| Gradient Boosting | 0.8085 | 0.7447 | 0.766 | 0.7234 | 0.7609 | 0.7607 |
| AdaBoost | 0.7021 | 0.6809 | 0.7021 | 0.6383 | 0.6304 | 0.6708 |
| XGBoost | 0.8085 | 0.7234 | 0.7447 | 0.7234 | 0.8043 | 0.7609 |
| LightGBM | 0.8085 | 0.7447 | 0.7447 | 0.7234 | 0.7174 | 0.7477 |
| CatBoost | 0.7872 | 0.766 | 0.8298 | 0.6809 | 0.8043 | 0.7736 |
