# Supplementary material for: Predicting depression among men who have sex with men in Ghana using machine learning algorithms
Source: PLOS Ment Health. 2025 Nov 20;2(11):e0000485. doi: 10.1371/journal.pmen.0000485 (PMC12798198; doi:10.1371/journal.pmen.0000485)
Supplement: S3 Table — These features reflect key psychosocial domains including perceived stress (PSS14), external social isolation (ExtSocialIso2), and stigma related to same-sex behavior (StigmaSSB9), among others influencing depression risk. (DOCX) [file pmen.0000485.s004.docx]

*S3 Table. Descriptions of the top features identified across tree-based machine learning models for predicting depression among MSM in Ghana. These features reflect key psychosocial domains, including perceived stress, social isolation, stigma, resilience, socioeconomic status, and community trust.*

| **Feature** | **Description** |
| --- | --- |
| ExtSocialIso2 | How often in the LAST WEEK did you meet face to face with friends and relatives living outside your household? |
| PSS14 | In the last month, how often have you felt difficulties were piling up so high that you could not overcome them? |
| StigmaSSB9 | How often have you lost a place to live because of being homosexual? |
| BRScale6 | I tend to take a long time to get over set-backs in my life. |
| PSS12 | In the last month, how often have you found yourself thinking about things that you have to accomplish? |
| SCS6 | People in my network of friends are good at influencing each other. |
| PSS3 | In the last month, how often have you felt nervous and “stressed”? |
| StigmaSSB6 | How often has your family not accepted you because of your homosexuality? |
| IntSocialIso5 | How strongly do you feel you belong to your immediate community/nei ghbourhood? |
| StigmaGNC2 | How often have you heard that men with feminine mannerisms are not normal? |
| SCS5 | I have a say about what goes on in my network of friends. |
| StigmaGNC12 | How often have you lost a job or career opportunity because of feminine mannerisms and behaviour? |
| Income_Category_Middle | How much money do you earn each month |
| IntSocialIso13 | If you lost a wallet that contained 800 cedis, and it was found by a neighbour, how likely is it to be returned with the |
