# Supplementary material for: Predicting depression among men who have sex with men in Ghana using machine learning algorithms
Source: PLOS Ment Health. 2025 Nov 20;2(11):e0000485. doi: 10.1371/journal.pmen.0000485 (PMC12798198; doi:10.1371/journal.pmen.0000485)
Supplement: Attachment — Submitted filename: Response to Reviewers.docx [file pmen.0000485.s007.docx]

**Manuscript Title**: Predicting Depression among Men Who Have Sex with Men in Ghana Using Machine Learning Algorithms
**Manuscript ID:** PMEN-D-25-00220
**Journal:** PLOS Mental Health
**Authors**: Abdulzeid Yen Anafo et al.
**Corresponding Author:** ayanafo@umat.edu.gh

**Response to Reviewer's Comments**

**Dear Editor and Reviewer,**

We sincerely thank you for the time and effort spent reviewing our manuscript. We appreciate the constructive feedback, which has helped us improve the clarity and overall contribution of our study. Below, we respond to each of the reviewer’s comments point-by-point. Reviewer comments are reproduced in bold, followed by our detailed responses in plain text. Changes made to the manuscript are clearly indicated in the revised version with tracked changes.

**Major Comments**

**Comment:** The study’s motivation is weakly developed. The objective of “detecting predictors” aligns more closely with traditional statistical analysis than with a machine learning use case. This is then conflated with “predicting depression” using ML. Why this prediction task is relevant, particularly in the context of interventions, screening, or policy, is barely touched. A clearer justification of the ML angle is needed.

**Response:** Thank you for this thoughtful comment. We acknowledge that the original manuscript did not sufficiently elaborate on the rationale for using machine learning (ML) over traditional statistical approaches, nor did it clearly articulate the relevance of predictive modelling within the context of public health interventions.

In response, we have revised the Introduction section to include a clearer justification for adopting ML. Specifically, we highlight that while statistical methods are adept at identifying associations, they often fall short in capturing non-linear relationships and high-dimensional interactions among risk factors an area where ML excels. Furthermore, we emphasise that predictive modelling has direct relevance for designing scalable, low-cost mental health screening tools that can support early detection and intervention among MSM in Ghana. This population often faces structural barriers to accessing care. These tools have the potential to inform targeted outreach and guide the allocation of limited mental health resources in both clinical and community settings.

**Comment:** The dataset comprises only 200 individuals, which is very limited for a machine learning study. There is increasing evidence that performance estimates based on such small samples carry a high risk of bias and offer limited generalizability. Additionally, most of the included algorithms (e.g., gradient boosting methods) are designed for large, high-dimensional datasets. It remains unclear how such mechanisms are expected to yield performance benefits on only 200 data points. At minimum, a strong justification for the chosen approach despite the sample limitations is required. Also, I believe a simpler baseline algorithm (e.g., penalized logistic regression) should be included for comparison.

**Response:**

Thank you for your insightful observation. We agree that the relatively small sample size of 225 individuals presents challenges for generalizability and performance evaluation in machine learning (ML). We have now taken the following steps to address this concern:

Justification for ML Use with a Small Dataset:
We have included an expanded explanation in the Discussion section highlighting that while ensemble algorithms like XGBoost and CatBoost are optimized for large datasets, they include regularization (e.g., L1/L2 penalties), early stopping, and shrinkage mechanisms that make them robust even in small-to-medium datasets. Our approach incorporated conservative model tuning, 5-fold cross-validation, and stratified train-test splits to reduce overfitting. We clarified that our goal is not only high performance but to assess the feasibility of deploying ML-based early screening models in low-resource contexts like Ghana, where data scarcity is common.

Addition of Penalized Logistic Regression as a Baseline:
As suggested, we implemented L1-regularized logistic regression (LASSO) as a baseline model. The model achieved a test accuracy of 75.6%, F1-score of 0.78, and ROC AUC of 0.82 comparable to the mid-tier tree-based models. These results are now reported in Table 2 and discussed in the Results and Discussion sections. Including this baseline highlights the performance gain from more complex models like Random Forest while also acknowledging that simpler interpretable models remain valuable.

Expanded Discussion on Generalizability:
We have explicitly acknowledged in the Limitations section that the findings may not generalize to broader MSM populations due to the modest sample size and recommend future work with larger, more representative datasets.

We thank the reviewer again for encouraging this critical addition, which has strengthened the methodological rigor and interpretability of our findings.

**Comment:** Especially for small samples, high methodological rigor is essential to avoid inflated and unreliable performance estimates. Unfortunately, the current study lacks transparency and shows signs of potential overfitting. I have the following more specific points

**Response**: We appreciate this important observation. Given the modest sample size, we agree that minimizing overfitting and ensuring transparency in model development are critical. In response, we have taken the following steps to enhance methodological rigor and clarity:

Cross-Validation Strategy Enhanced and Clarified:
We now clarify that a stratified 5-fold cross-validation approach was applied throughout model development. This ensures that each fold preserved the proportion of depressed vs. non-depressed cases, minimizing sampling bias. The mean performance metrics reported reflect these cross-validation results, and we have clearly distinguished between cross-validation and test set results in both the Results and Tables 1–2.

Model Overfitting Prevention Techniques:

All models were trained with early stopping where applicable.

Default hyperparameters were adjusted cautiously, avoiding over-tuning.

SMOTE was applied only on the training folds, not on the full dataset, to avoid data leakage.

Feature engineering was limited to binning and standardization based on prior theory, not automatic transformation.

Transparency Improvements:

A complete breakdown of model performance (train vs. test) is now provided in the updated Results section.

All preprocessing steps, feature transformations, and evaluation metrics have been described in detail in the Methods section.

We now include the code used for model development and evaluation in the supplementary material (or upon request, depending on your journal’s policy).

Model Comparison Expanded:
To provide context for model performance and reduce the impression of inflated results, we added a penalized logistic regression model as a simpler baseline. Its performance, now presented alongside the ensemble models, helps calibrate expectations and validates the improvements obtained using more complex classifiers.

We believe these revisions meaningfully improve the study’s transparency and reliability and hope they adequately address the concern regarding potential overfitting and lack of methodological detail.

**Comment:** Code availability-Please make the full analysis code publicly available (e.g., via GitHub). This is essential for reproducibility and enables reviewers and readers to assess several unclear methodological aspects.

**Response:** Thank you for this important suggestion. We agree that making the analysis code publicly available enhances transparency, reproducibility, and the overall integrity of the study. In response, we have now uploaded the complete analysis scripts, including preprocessing, model development, performance evaluation, and feature importance plots, to a public GitHub repository. The code repository is available at: https://github.com/Zeidyen/msm-depression-ml

**Comment:** Data preprocessing-It appears that imputation and over-/undersampling were conducted prior to the train/test split. If this interpretation is correct, this constitutes data leakage and is methodologically incorrect. Please clarify the order of preprocessing steps.

**Response:** We thank the reviewer for pointing out this critical issue. We fully agree that performing imputation or resampling before the train/test split would introduce data leakage and compromise model validity.

We clarify that in our analysis:

The dataset was first split into training (80%) and testing (20%) subsets using stratified sampling to preserve class proportions.

All imputation (median filling of missing values) and SMOTE oversampling were applied only to the training set during the preprocessing pipeline.

The test set remained untouched by imputation or resampling until final evaluation to simulate a real-world, unseen data scenario.

Standardization and one-hot encoding were fitted on the training set and then applied to the test set using the same fitted scalers/encoders.

**Comment:** Train/test split explanation-The description of the train/test split is very difficult to follow. The manuscript mentions both a 5-fold cross-validation and an 80/20 split—this needs to be clarified and unambiguously described.

**Response:** Thank you for this important observation. We acknowledge that our original explanation may have been confusing regarding the relationship between the 80/20 train-test split and the use of 5-fold cross-validation.

**Comment:** Performance variation-With such a small dataset, performance estimates are likely to vary significantly depending on random initializations or data splits or algorithms. Please report the variation (e.g., standard deviation) across repetitions. Without this, any interpretation of algorithm performance differences remains speculative.

**Response:** We appreciate the reviewer’s important point regarding performance variability, especially with a small dataset. While we acknowledge that reporting standard deviations can provide a deeper understanding of the robustness of model performance, we chose to maintain a more streamlined evaluation approach for this initial study for the following reasons:

Focus on Feasibility and Benchmarking:
The goal of this study is to explore the feasibility of applying machine learning models to predict depression risk in a low-resource context and to benchmark different algorithms. Accordingly, we opted for a single stratified 5-fold cross-validation scheme, which is widely used and balances computational tractability with robustness.

To ensure consistency and reduce random variability:

We set fixed random seeds for data splits and model training.

We used stratified sampling for the 80/20 train-test split and within cross-validation folds to preserve outcome distribution.

The same data partitions were applied across all models for a fair comparison.

Transparent Limitations Acknowledged:
We have now expanded the Limitations section to explicitly acknowledge that performance estimates may vary due to the sample size and randomness inherent to machine learning. We have also stated that future studies with larger samples should incorporate repeated cross-validation or bootstrapping to capture uncertainty in model performance better. We hope this explanation provides sufficient justification for our chosen evaluation approach while acknowledging the limitations, and we thank the reviewer again for prompting this important clarification.

**Comment:** Hyperparameter tuning-What method was used for hyperparameter tuning? Please describe this clearly.

**Response:** We thank the reviewer for highlighting the need for clarity regarding hyperparameter tuning.

In the current study, we used default parameters for all classifiers to establish baseline comparisons among tree-based models. This choice was intentional, as our goal was to assess the feasibility of applying various machine learning models for depression risk prediction in a small dataset, rather than to maximize performance for a single model through extensive tuning. To clarify this in the manuscript, we have added the following statement to the Methods.

**Comment**: Why was accuracy chosen as the main evaluation metric? How was the threshold for binary classification determined? If the default of 0.5 was used, why? The same question applies to other threshold-dependent metrics such as recall.

**Response:** Thank you for raising this important concern. We acknowledge the limitations of using accuracy as the sole metric, particularly with imbalanced datasets. To address this, we complemented accuracy with threshold-dependent metrics such as precision, recall, and F1-score, as well as the ROC-AUC, which is threshold-independent and provides a more balanced assessment.

For classification, we used the default probability threshold of 0.5 for binary classification, as implemented in the respective Scikit-learn classifiers. This default threshold allows for consistent comparison across models. Given the study’s aim to compare baseline performance across multiple tree-based classifiers, we prioritized interpretability and reproducibility over extensive threshold tuning.

**Comment:** The reported AUC of 0.91 raises serious concerns about overfitting. Is there a credible explanation for how depression could be predicted so accurately from sociodemographic variables alone? If the authors believe this result is valid, it should be discussed in depth—particularly in comparison to established findings from high-quality studies using similar data and targets.

**Response:** We appreciate the reviewer’s critical observation regarding the high AUC (0.91). We fully agree that such a result should be interpreted with caution, particularly given the limited sample size and the use of non-clinical features. Several factors may have contributed to this high AUC: First, the model was trained using well-established psychosocial predictors of depression, such as Perceived Stress Scale (PSS) items and social isolation metrics, which are known to correlate strongly with depressive symptoms, especially among marginalized populations like MSM in Ghana. Second, the outcome variable (PHQ-2) is closely related to these psychosocial inputs, potentially inflating internal predictive accuracy. Third, performance was assessed using stratified cross-validation and evaluated on a held-out test set. However, due to the small dataset size, some degree of overfitting remains a possibility, despite methodological safeguards like SMOTE and standardization.

To address this concern, we have included additional discussion in the revised manuscript that: Acknowledges the potential for overfitting and the lack of external validation, compares our findings with prior studies using similar psychosocial data for depression prediction and Emphasizes the exploratory nature of the study and the need for validation in larger, independent cohorts before generalizing the model’s utility.

**Comment:** The method used to compute feature importance is not explained. Please clarify.
Response: We thank the reviewer for this valuable observation. In response, we have revised the manuscript to include a clear explanation of how feature importance was derived.

**Minor Comments**

**Comment :** Abstract: The abstract is weak. Technical details of the ML pipeline do not belong in the abstract. More essential information is missing, such as the sample size.
**Response:** Thank you for this insightful comment. We have revised the abstract to enhance clarity for a broader audience.

**Comment:** The introduction lacks a clear structure. I do not understand the relevance of studies on social media-based classification, which are only loosely related to the current approach.

**Response:** Thank you for this insightful comment. We have revised the introduction section to enhance clarity for a broader audience.

**Comment:** The feature selection method is not clearly explained. Please clarify how features were selected and what rationale guided the process.

**Response:** Thank you for this insightful comment. We have revised the Feature Selection section to enhance clarity.

We thank the reviewer and editorial team again for their helpful insights. We look forward to your feedback on the revised version.

Sincerely,
**Abdulzeid Yen Anafo, PhD**
(On behalf of all co-authors)
