# Supplementary material for: Predicting depression among men who have sex with men in Ghana using machine learning algorithms
Source: PLOS Ment Health. 2025 Nov 20;2(11):e0000485. doi: 10.1371/journal.pmen.0000485 (PMC12798198; doi:10.1371/journal.pmen.0000485)
Supplement: Attachment — Submitted filename: Reviewer_response.docx [file pmen.0000485.s008.docx]

**Response to reviewers**

**Reviewer #2:** This study focused on applying machine learning models to identify predictors of depression in a sample of 225 men who have sex with men in Ghana.

**Comments:** Consider: "The full dataset was initially partitioned into training (80%) and testing (20%) sets using stratified sampling to maintain the distribution of the target variable. All data preprocessing steps (imputation, SMOTE, encoding, and scaling) were applied only to the training set to prevent data leakage. The training set was then used to develop the models and tune hyperparameters using stratified 5-fold cross-validation. Model performance was evaluated on the test set, which remained untouched throughout the training process.";
This text is very confusing:
a) It says 80/20 and, after that, 5-fold cross-validation. Is it nested cross-validation? If yes, make it clear.
b) To work, data preprocessing steps (exception for SMOTE) must be 'applied' to the test dataset too. Perhaps the authors would like to say that they were defined/calculated based on the training dataset.
c) It's not clear why you needed to apply SMOTE. What is the original/initial class balance?

**Responses:** 1a) 80/20 split and 5-fold CV—nested CV?
Thank you for the comment. We did not use nested CV. We held out a single 20% test set and performed stratified 5-fold CV only within the 80% training data for model selection and tuning. We have revised the Methods to state this explicitly ***(see Data Pre-processing and Partitioning section Page 7 lines 205-213)***

1b) “Applied only to the training set.”
Thank you. We agree that our phrasing could be clearer. We now state that transformers were fit (parameters learned) on training data only and then applied (transform) to validation folds and the test set using those parameters. SMOTE was never applied to validation or test data; it was restricted to the training portion within each CV split. The Methods have been updated accordingly. ***(see Handling Class Imbalance section Page 7 lines 231-234)***

1c) Why SMOTE? What was the initial class balance?
We have added the baseline class distribution in the Methods. Given this imbalance, SMOTE was used only during model fitting on training folds to reduce majority-class bias and improve sensitivity to the minority class, while preserving a realistic distribution in validation and test sets. We also report metrics beyond accuracy (ROC-AUC, F1, recall, precision) to reflect performance under imbalance. (***see Handling Class Imbalance section Page 7 lines 231-234)*** )

**Comment 2:** What is 'error rate'? I think it is the same than accuracy.
**Response:** Thank you for the comment. We did not report error rate. We reported accuracy, and for clarity we have taken the error rate out in ***page 8 between lines 240-241***

**Comment 3:** There is no reason to explain the ML models in different (sub)sections.

**Response:** Thank you for the comment. ML models have all been put into one subsection. ***Page 9 between lines 251-275.***

**Comment 4:** ROC curve of the decision tree model (figure 1) looks wrong.
**Response:** Thank you for the observation. We regenerated the ROC for the Decision Tree using scikit-learn with predicted probabilities for the positive class and an explicit pos_label, and obtained the same AUC (approximately 0.66). The staircase appearance is somewhat expected as a single CART produces only a small set of distinct probability values (one per leaf), and with a modest test size, this yields large steps. The curve is monotone from (0,0) to (1,1) and the AUC matches the numeric result, indicating the plot is correct.

**Comment 5:** When analysing results in sections 'Classification report on training data' and 'Model evaluation on test data', they seem not to present any overfitting. Do you agree?

**Response:** Yes, we agree our results do not indicate overfitting. Performance on the held-out test set closely matches the mean cross-validated performance on the training data across models. Also, the test-set confusion matrices show both classes are being identified, and precision/recall/F1 remain aligned with CV results.

**Comment 6:** There is no reason to state "Future work involving larger and independent datasets will explore model optimization techniques more extensively" in the methods section.

**Response:** The statement “Future work involving larger and independent datasets will explore model optimization techniques more extensively” has been removed from the methods section.

**Comment 7:** Remove "For tree-based models (Decision Tree, Random Forest, Gradient Boosting, AdaBoost, XGBoost, LightGBM, and CatBoost)" - lines 223-224; all models are based on tree.

**Response:** Thank you for the comment. We have removed the term “tree-based models” ***Page 7 Lines 207-211.***

**Comment 8**: Here is your main conclusion: "The feature importance analysis revealed that social isolation, perceived stress, and trust in others were the most consistent and influential predictors of depression."; have you checked how the models work using only these three variables as predictors?

**Response:** Thank you for the comment. We conducted a reduced-feature (three-predictor) sensitivity analysis using only social isolation, perceived stress, and trust/belonging (operationalised as ExtSocialIso2, a PSS stress measure, and IntSocialIso13) within the same pipeline Random Forest with only these three predictors retained a meaningful signal but underperformed the full-feature model, while a logistic baseline performed lower still. These findings support our conclusion that the three domains are highly influential yet insufficient alone; additional features provide complementary information that enhances discrimination.

We have updated the manuscript accordingly: ***Methods (p. 10, lines 278–285),*** ***Results (p. 15, lines 379–388)***, ***Discussion (p. 17, lines 464–469),*** and we have revised the Conclusion to reflect these additions.

**Comment 9:** Now, I'll focus on a previous comment from another reviewer and authors' reply.
- Consider "Comment: Hyperparameter tuning-What method was used for hyperparameter tuning? Please describe this clearly.
Response: We thank the reviewer for highlighting the need for clarity regarding hyperparameter tuning. In the current study, we used default parameters for all classifiers to establish baseline comparisons among tree-based models. This choice was intentional, as our goal was to assess the feasibility of applying various machine learning models for depression risk prediction in a small dataset, rather than to maximise performance for a single model through extensive tuning.";
- This is conflicting, because you provide the following information in the manuscript "The training set was used for model training and hyperparameter tuning, while the test set was reserved for performance evaluation on unseen data."
**Response:** Thank you for noting the inconsistency. Our study employed pre-specified (untuned) hyperparameters to provide fair and reproducible baseline comparisons across tree-based models; we did not perform hyperparameter tuning. We have revised the Methods accordingly, ***Page 7, Lines 212-213***. We also note this choice on the Limitations ***Page18, lines 505-507***, emphasising that our goal was feasibility and comparability rather than model-specific optimisation.
